# Noise-Driven Phenotypic Heterogeneity with Finite Correlation Time in Clonal Populations

**DOI:** 10.1371/journal.pone.0132397

**Published:** 2015-07-23

**Authors:** UnJin Lee, John J. Skinner, John Reinitz, Marsha Rich Rosner, Eun-Jin Kim

**Affiliations:** 1 Committee on Genetics, Genomics, and Systems Biology, University of Chicago, Chicago, IL, United States of America; 2 Ben May Department for Cancer Research, University of Chicago, Chicago, IL, United States of America; 3 Department of Biochemistry and Molecular Biology, University of Chicago, Chicago, IL, United States of America; 4 Departments of Statistics, Ecology and Evolution, Molecular Genetics and Cell Biology, University of Chicago, Chicago, IL, United States of America; 5 School of Mathematics and Statistics, University of Sheffield, Sheffield, United Kingdom; University of Southampton, UNITED KINGDOM

## Abstract

There has been increasing awareness in the wider biological community of the role of clonal phenotypic heterogeneity in playing key roles in phenomena such as cellular bet-hedging and decision making, as in the case of the phage-λ lysis/lysogeny and *B. Subtilis* competence/vegetative pathways. Here, we report on the effect of stochasticity in growth rate, cellular memory/intermittency, and its relation to phenotypic heterogeneity. We first present a linear stochastic differential model with finite auto-correlation time, where a randomly fluctuating growth rate with a negative average is shown to result in exponential growth for sufficiently large fluctuations in growth rate. We then present a non-linear stochastic self-regulation model where the loss of coherent self-regulation and an increase in noise can induce a shift from bounded to unbounded growth. An important consequence of these models is that while the average change in phenotype may not differ for various parameter sets, the variance of the resulting distributions may considerably change. This demonstrates the necessity of understanding the influence of variance and heterogeneity within seemingly identical clonal populations, while providing a mechanism for varying functional consequences of such heterogeneity. Our results highlight the importance of a paradigm shift from a deterministic to a probabilistic view of clonality in understanding selection as an optimization problem on noise-driven processes, resulting in a wide range of biological implications, from robustness to environmental stress to the development of drug resistance.

## Introduction

With the advent of new technologies allowing for precise, quantitative measurement of protein levels at cellular resolution, there has been greater awareness in the wider biological community challenging the assumption that all observed experimental noise in biological systems is an inherent artifact of experimental processes. Rather, randomly varying elements inherent to individual cells, such as fluctuating protein levels [1], contribute to cell-to-cell heterogeneity and are key contributors to processes that control a variety of biological phenomenon on multiple scales, ranging from cellular decision-making [2, 3], to the evolutionary fitness of monoclonal yeast colonies [4]. A recent essay [5] highlighted the key role of non-heritable cellular variability in the evolution processes of cancer.

Stochasticity is inherent in any well mixed chemical solution [6]. Therefore noise will exist in biological systems regardless of how precisely these systems can be measured. Furthermore, as there are always factors that are inaccessible to observers at any given resolution, taking into account all relevant physical/biochemical processes when modeling biological systems across multiple scales is practically impossible. The totality of these processes are treated here as stochastic noise, covering many biological, chemical, and physical processes including epigenetic and environmental effects. In this paper, we model noise-driven growth in biological systems in the scenario of simple multiplicative noise as well as that of a first-order self-regulation. It is important to note that the noise considered here always has a mean value of zero. As such, random deviations from mean growth rates make mean values inadequate representations of our system, necessitating a probabilistic approach by using probability distribution functions (e.g. see [7, 8]). The clonal populations described here have identical genetic compositions and therefore identical model parameters, but reside in varying areas of state-space due to the stochasticity of the system. Such phenomena have been observed in the form of multiple, discrete colonies of yeast, where it has been posited that such phenotypic variability provides a mechanism for evolutionary bet-hedging [4]. We introduce a mathematical formalism that can describe bet-hedging and other similar phenomena.

## 1 Models

We model the consequences of noise driven growth as solutions to stochastic differential equations (SDEs) in the form of probability distribution functions (PDFs), thereby highlighting the necessity of probabilistic approaches when understanding the issue of cellular heterogeneity within clonal sub-populations. In general, stochastic differential equations contain both a deterministic and stochastic component, where the stochastic component is written as the product of a deterministic coefficient and a Gaussian noise with zero mean. In our models, we consider two types of Gaussian noise—Gaussian white noise and Gaussian colored noise. An important distinction between the two is that white noise has no intrinsic memory, while colored noise has a finite auto-correlation time. Gaussian white noise (short correlation time *τ*_*c*_), *g*_*w*_(*t*), is represented as $g_{w}(t)=\frac{dW}{dt}$ with the property 〈*g*_*w*_(*t*)*g*_*w*_(*t*′)〉 = *Dτ*_*c*_ *δ*(∣*t* − *t*′∣), where *W* is a continuous-time Wiener process and *δ* is a delta function. Gaussian colored noise, *g*_*c*_(*t*), can be described as the solution to the equation

$$\frac{dg_{c}\left(t\right)}{dt}=-\tau_{c}g_{c}\left(t\right)+\tau_{c}g_{w}\left(t\right),$$ (1)

with the auto-correlation property

$$\left\langle g_{c}\left(t\right)g_{c}\left(t^{\prime }\right)\right\rangle =De^{{}^{-t}\;{/}_{\tau_{c}}},$$ (2)

where *τ*_*c*_ is the auto-correlation time and *D* is the magnitude of noise (S1 Fig). It is important to note that both *g*_*w*_(*t*) and *g*_*c*_(*t*) both share identical means and variances whose distributions cannot be distinguished at any single point in time. Auto-correlated noise, similar to that described Eq 2, has been previously observed as a driving factor in biological systems [1, 9].

In our models, intermittency, or a stochastic change in state, is introduced by way of the finite auto-correlation time. While the trajectories of the Gaussian colored noise cannot be considered as Markov processes, we can highlight the essential effect of the auto-correlation time *τ*_*c*_ by decomposing our random process into a two-state Markov process. By assigning negative values of *g*_*w*_(*t*) and *g*_*c*_(*t*) to state 0 and positive values of *g*_*w*_(*t*) and *g*_*c*_(*t*) to state 1, we can see that an increase of the auto-correlation time, *τ*_*c*_, would result in an increase of the 0 → 0 and 1 → 1 transition probability, thereby resulting in a net increase in intermittency as shown by a decreased probability of state transitions. (S2 Fig) In the context of our linear model, the decomposition of positive and negative values to binary states can be interpreted as either decay (state 0) or growth (state 1). To clarify, sub-populations of the linear model can be either growing or decaying, with transitions between each state being stochastic. As such, these systems are intermittent. With lower transition probabilities, the expected times between state transitions increase and thus have higher intermittency.

### 1.1 Stochastic Differential Equations

The models presented in the following section are solutions to the stochastic differential equation

$$\frac{dC_{l}}{dt}=g_{c}C_{l},$$ (3)

for the standard multiplicative noise scenario, referred to as our linear model. Similar models have been previously proposed for describing diverse biological phenomena, from the survival times of cancer patients to spine sizes in the neocortex [10, 11]. Further studies of this linear model have also been previously described for similar colored noise regimes [12].

We also present our non-linear model as the coupled SDEs

$$\frac{dC_{n}}{dt}=GC_{n},$$ (4)

and

$$\frac{dG}{dt}=\gamma -\eta C_{n}-G,$$ (5)

where *C*_*l*_ (linear) and *C*_*n*_ (non-linear) are a continuous valued functions representing cellular populations, G represents the overall growth input from various molecular pathways, *γ* is a constitutive input of growth, and *η* is a reflection of self-regulation of *G* by *C*_*n*_. The overall effect of self-regulation of the total growth input is described as

$$\eta =\epsilon +g_{c}\left(t\right),$$ (6)

where *ϵ* is a positive constant describing the degree of coherent self-regulation.

For the statistically steady state, $\frac{dG}{dt}∼0$, we model a loss of coherent population-based self-regulation by proposing the following generalized logistic-type model

$$G∼\gamma -\left(\epsilon +g_{c}\right)C_{n},$$ (7)

where Eq 4 becomes

$$\frac{dC_{n}}{dt}=\gamma C_{n}-\left(\epsilon +g_{c}\right)C_{n}^{2}.$$ (8)

### 1.2 Solutions

#### PDFs

As the value of *C*_*l*_ changes stochastically with a stochastic noise *g*_*c*_, we quantify the evolution of *C*_*l*_ by the PDF of *C*_*l*_, denoted by *P*(*C*_*l*_). Interestingly, to the first order in *τ*_*c*_, Eq 3 gives the Fokker-Planck equation for *P*(*C*_*l*_) where the effect of *τ*_*c*_ disappears (c.f. [13]). In order to capture the effect of *τ*_*c*_ without using a small *τ*_*c*_ approximation, we derive *P*(*C*_*l*_) directly from Eqs 2 and 3. To this end, we express Eq 3 in terms of $\Gamma (t)=\int_{0}^{t}g_{c}(t_{1})dt_{1}$ to obtain.

$$\ln\frac{C_{l}}{C_{0}}=\Gamma ,$$ (9)

where *C*_0_ = *C*_*l*_(*t* = 0) is the initial value of *C*_*l*_ at *t* = 0. Eq 9 gives *d*Γ/*dC*_*l*_ = 1/*C*_*l*_, which is then used to link the PDFs of *C*_*l*_ and Γ as follows:

$$P\left(C_{l}\right)=P\left(\Gamma \right)\frac{d\Gamma }{dC_{l}}=\frac{1}{C_{l}}P\left(\Gamma \right).$$ (10)

*P*(Γ) in Eq 12 can be computed by using its characteristic function 〈*e* ^*ik*Γ^〉 as:

$$\begin{array}{ccc} P(\Gamma ) & = & \frac{1}{2\pi }\;\int dke^{-ik\Gamma }\left\langle e^{ik\Gamma }\right\rangle \\ & = & \frac{1}{2\pi }\;\int dke^{-ik\Gamma }e^{-k^{2}\left\langle \Gamma^{2}\right\rangle /2}. \end{array}$$ (11)

〈Γ^2^〉 in Eq 11 is obtained by using Eq 2 as:

$$\begin{array}{ccc} \left\langle \Gamma^{2}\right\rangle & = & \int_{0}^{t}dt_{1}\;\int_{0}^{t}dt_{2}\left\langle g_{c}\left(t_{1}\right)g_{c}\left(t_{2}\right)\right\rangle \\ & = & 2D\;\int_{0}^{t}dt_{1}\;\int_{0}^{t_{1}}dt_{2}e^{\left[-\left(t_{1}-t_{2}\right)/\tau_{c}\right]}\\ & = & 2D\tau_{c}\;[t-\tau_{c}\left(1-e^{-t/\tau_{c}}\right)]. \end{array}$$ (12)

By performing the integral over *k* in Eq 11 by using Eq 12 and substituting it to Eq 10, we obtain the solution to Eq 3 as described by the probability distribution function

$$P\left(C_{l}\right)=\frac{1}{2C_{l}\sqrt{\alpha t\pi }}\exp\left[-\frac{{\left[\ln C_{l}/C_{0}\right]}^{2}}{4\alpha t}\right].$$ (13)

where

$$\alpha =D\tau_{c}\;[1-\frac{\tau_{c}}{t}\left(1-e^{-t/\tau_{c}}\right)].$$

Note that in the limit of a short correlation time *τ*_*c*_ → 0, *α* → *Dτ*_*c*_

**Fig 1:**
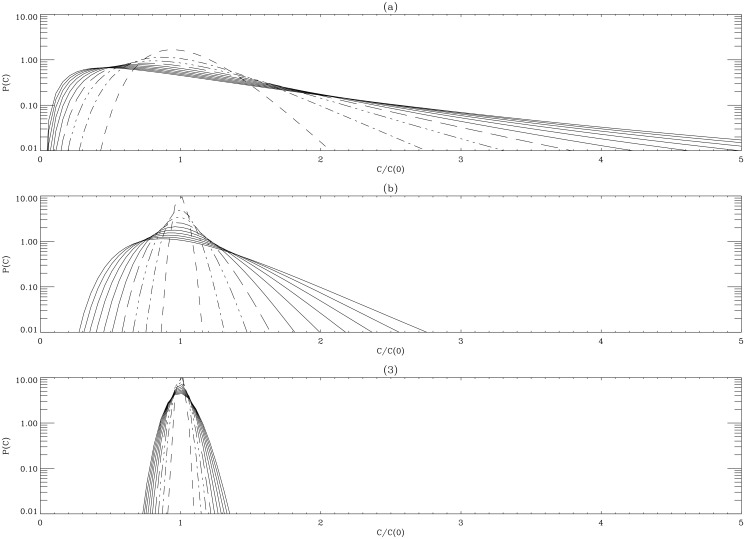
Variability of population is influenced by both the magnitude and auto-correlation properties of noise. Time evolution of *P*(*C*_*l*_, *t*) shown at *t* = 0.001 + 0.4*n* (*n* = 0, 1, 2, …10) for *C*_0_ = 1. *τ*_*c*_ = 0.01 and *D* = 100 in panel (A); *τ*_*c*_ = 1 and *D* = 1 in panel (B); *τ*_*c*_ = 0.01 and *D* = 1 in panel (C). Time increases from narrower to wider distributions in each panel.

Here, we recover a log-normal distribution, as is expected for stochastic systems driven solely by Gaussian multiplicative noise. To illustrate this, we show P(*C*_*l*_, t) for three different values of *τ*_*c*_ and *D* (Fig 1). In doing this, we keep the same value for *τ*_*c*_ *D* since taking the limit of short correlation time requires keeping *τ*_*c*_ *D* constant. Specifically, (Fig 1a and 1b) are for *τ*_*c*_ = 0.01 and *D* = 100 and for *τ*_*c*_ = 1 and *D* = 1, respectively. For the initial delta-function PDF, *P*(*C*_0_, 0) = *δ*(*C*_0_ − 1) (*C*_0_ = *C*_*l*_(0)), PDFs are shown at *t* = 0.0001 + 0.4*n* where *n* = 0, 1, 2, …10. In both cases, we can see that as time increases, the peak of the PDF moves towards the value *C*_*l*_ = 0 while the right PDF tail stretches out to larger values of *C*_*l*_ with its height increasing with time. This stretched PDF is due to colonies of large population size increasing in time, and is a manifestation of intermittency. It is important to note that the growth rates of each colony is uniquely determined and non-synchronous. The comparison between Fig 1a and 1b reveals that the evolution of the PDF with a longer correlation time *τ*_*c*_ = 1 in Fig 1b keeps its original PDF longer, with its peak around *C*_*l*_ = 1, demonstrating the dynamic relationship between correlation time and dispersion.

This result demonstrates an intrinsic trade-off between accessible state-space and growth stability, which may be of particular relevance during recovery after a given selection sweep. After a selective sweep, remaining survivors are selected by virtue of having adopted a selectively favorable state during the application of selection. Notably, survivors can either be statically or transiently in such a selectively favorable state While continuously residing in the neighborhood of a particular state may be advantageous for a small range of selective sweeps at many time points, transiently residing in a wider range of states can be thought of as conferring fitness advantages for a larger range of selective sweeps, though only for only a select amount of time. For large clonal populations composed of many discrete sub-populations or colonies, the latter scenario allows for an overall larger range of states to be simultaneously accessed. Thus, in anticipation for a wide range of potential sweeps, it may be advantageous for certain populations to adopt such bet-hedging strategies that may increase overall variability, allowing for an increased probability of survival. However, such bet-hedging strategies are constrained by inherent growth instabilities that may arise due to excess variability.

To derive the solutions to Eq 8, we first compute the PDF of population *P*(*C*_*n*_). To this end, we recast Eq 8 in terms of *y* = 1/*C*_*n*_ as follows:

$$\frac{dy}{dt}=\gamma y-\epsilon +g_{c}.$$ (14)

The solution to Eq (14) is then found as:

$$y-y\left(0\right)e^{-\gamma t}-\frac{\epsilon }{\gamma }\left[1-e^{-\gamma t}\right]=\int_{0}^{t}dt_{1}e^{-\gamma (t-t_{1})}g_{c}\left(t_{1}\right)\equiv z,$$ (15)

where *y*(0) = 1/*C*(0) is the initial value of *y* at *t* = 0. The PDF of *z* can then be obtained from its characteristic function, following a similar analysis as for *C*_*l*_:

$$\begin{array}{ccc} P(z) & = & \frac{1}{2\pi }\;\int dke^{-ikz}\left\langle e^{ikz}\right\rangle \\ & = & \frac{1}{2\pi }\;\int dke^{-ikz}e^{-k^{2}\left\langle z^{2}\right\rangle /2}, \end{array}$$ (16)

where 〈*z* ^2^〉 in Eq (16) is obtained by using Eq (2) as:

$$\begin{array}{ccc} \left\langle z^{2}\right\rangle & = & \int_{0}^{t}dt_{1}\;\int_{0}^{t}dt_{2}e^{-\gamma \left(t-t_{1}\right)-\gamma \left(t-t_{2}\right)}\left\langle g_{c}\left(t_{1}\right)g_{c}\left(t_{2}\right)\right\rangle \\ & = & \frac{D}{\gamma }\;[\frac{1}{\gamma +\tau_{c}^{-1}}-\frac{2\gamma }{\gamma^{2}-\tau_{c}^{-2}}e^{-(\gamma +\tau_{c}^{-1})t}+\frac{1}{\gamma -\tau_{c}^{-1}}e^{-2\gamma t}]. \end{array}$$ (17)

The substitution of Eq (17) in Eq (16) and the evaluation of the Gaussian integral then gives us

$$P\left(C_{n}\right)=\frac{N}{C_{n}^{2}}\exp\left[-\beta \;{\left[\frac{1}{C_{n}}-\frac{e^{-\gamma t}}{C\left(0\right)}-\frac{\epsilon }{\gamma }\left(1-e^{-\gamma t}\right)\right]}^{2}\right].$$ (18)

Here $N^{-1}=\int_{-A}^{\infty }dze^{-\beta z^{2}}$ is the normalization factor, $A=\frac{e^{-\gamma t}}{C(0)}+\frac{\epsilon }{\gamma }(1-e^{-\gamma t})$, *β* = (2〈*z* ^2^〉)^−1^ and $P(C_{n})=P(z)/C_{n}^{2}$ were used.

It must be noted here as well that the driving noise, *g*_*c*_, is determined uniquely for each colony and is non-synchronous. These solutions have been computationally validated using a stochastic Runge-Kutta method for Gaussian colored noise (S3 and S4 Figs and S1 Text) [14].

**Fig 2:**
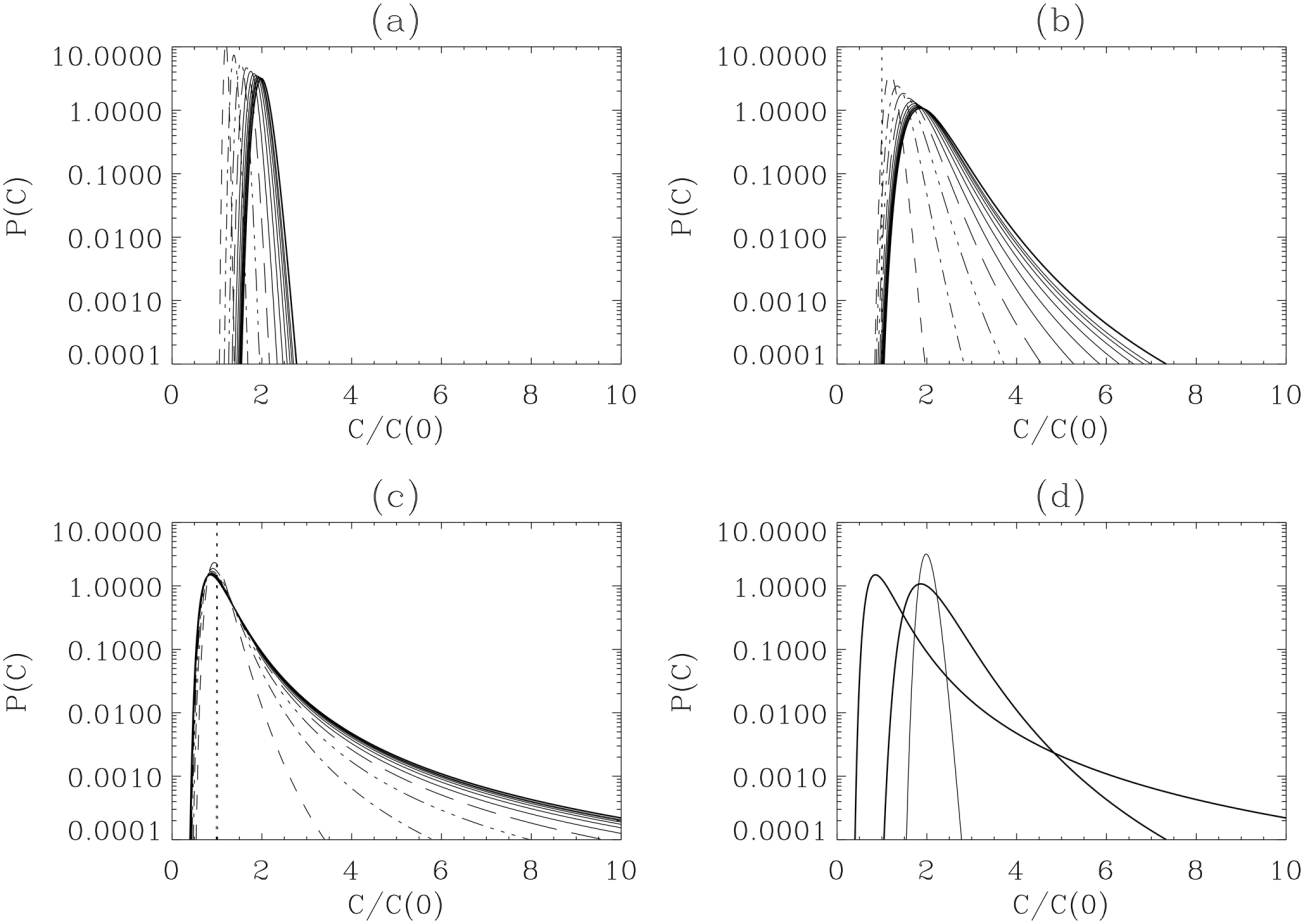
*P*(*C*_*n*_, *t*) converges on a stationary distribution. *P*(*C*_*n*_, *t*) for *C*_0_ = 1 and *t* = 0.001 + 0.4*n* where *n* = 0, 1, 2, 3, …10 for the parameter values of *γ* = 1, *ϵ* = 0.5, *β* = 500 [panel (A)]; *γ* = 1, *ϵ* = 0.5, *β* = 50 [panel (B)]; *γ* = 0.5, *ϵ* = 0.5, *β* = 5 [panel (C)]. The initial PDF *P*(*C*_0_, 0) = *δ*(*C*_0_ − 1) is shown in a vertical dotted line; the stationary PDF at *t* = 4 is shown in a thick solid line in panels (A)-(C). Stationary PDFs in panels (A)-(C) are superimposed in panel (D).

To understand how this system grows in general, we show *P*(*C*_*n*_) at different times *t* = 0.001 + 0.4*n* (where *n* = 0, 1, 2, 3, …, 9, 10) for fixed values of *C*_0_ = 1. The three cases shown in Fig 2(a)–2(c) are for the parameter values:

- Fig 2(a): *γ* = 1, *ϵ* = 0.5, *β* = 500 (C_*_ = 2);
- Fig 2(b): *γ* = 1, *ϵ* = 0.5, *β* = 50 (C_*_ = 2);
- Fig 2(c): *γ* = 0.5, *ϵ* = 0.5, *β* = 5 (C_*_ = 1).

The first two cases have the same carrying capacity C_*_ = *γ*/*ϵ* = 2 while the strength of stochastic noise increases from Fig 2a and 2b as *β* decreases from 500 to 50. For the case with the smallest stochastic noise *β* = 500 in Fig 2a, the PDF starting from a delta-function at *C*_*n*_ = 1 (shown in a dotted vertical line) moves slowly to C_*_ = 2, with little broadening of its width, and converges to a stationary PDF plotted as a thick solid line, with a peak forming around its carrying capacity C_*_ = 2. When *β* decreases from 500 to 50 by a factor of 10 as the stochastic noise increases by a factor of 10, the PDF becomes broader, and a long tail develops on the right (large *C*_*n*_) and also somewhat on the left (small *C*_*n*_). This is shown in Fig 2b, which is taken at the same times as in Fig 2a.

A further decrease in *β* by a factor of 5 not only broadens the PDF further, but also causes a shift of the PDF peak to a smaller value of *C*_*n*_ < C_*_. This case however is not shown in Fig 2. Instead, as both *γ* and *g*_*c*_ act to promote growth, in Fig 2c, we show the case where *γ* is reduced by a factor of 2 when *β* is further reduced (i.e. for larger *g*_*c*_). Taken at the same times as in Fig 2a and 2b, Fig 2c shows a broader distribution with a heavier right PDF tail. Stationary PDFs in Fig 2a–2c are superimposed in Fig 2d to facilitate the comparison among the three cases. It is notable that the right PDF tail is much heavier than the left PDF tail, with skewness increasing with the loss of self-regulation.

#### Mean and Variance

From the movement of the peaks of our PDFs alone, it is not entirely clear if the average increases or decreases in time. For the linear model Eq 3, we thus compute the average and also the dispersion of *C*_*l*_ as follows.

$$\left\langle C_{l}\right\rangle =C_{0}e^{\alpha t}$$ (19)

$$\Delta C_{l}=\sqrt{\left\langle C_{l}^{2}\right\rangle -{\left\langle C_{l}\right\rangle }^{2}}=C_{0}\sqrt{e^{4\alpha t}-e^{2\alpha t}}\approx C_{0}e^{2\alpha t},$$ (20)

where again *C*_0_ = *C*_*l*_(*t* = 0) is the initial value of *C*_*l*_ at *t* = 0 (additional derivations for these quantities can be found in S1 Text). Thus, despite the movement of the peak of the PDFs towards smaller values, Eq 19 reveals that a system driven by purely stochastic noise grows exponentially in time with a time-varying growth rate $\alpha =D\tau_{c}\;\left[1-\frac{\tau_{c}}{t}(1-e^{\frac{-t}{\tau_{c}}})\right]$. This is due to the contribution from the heavy PDF tail, as noted above. Eq 20 also shows that the dispersion Δ*C*_*l*_ grows at an even faster rate 2*α*, signifying a rapid growth of intermittency (heterogeneity) as the system progresses.

**Fig 3:**
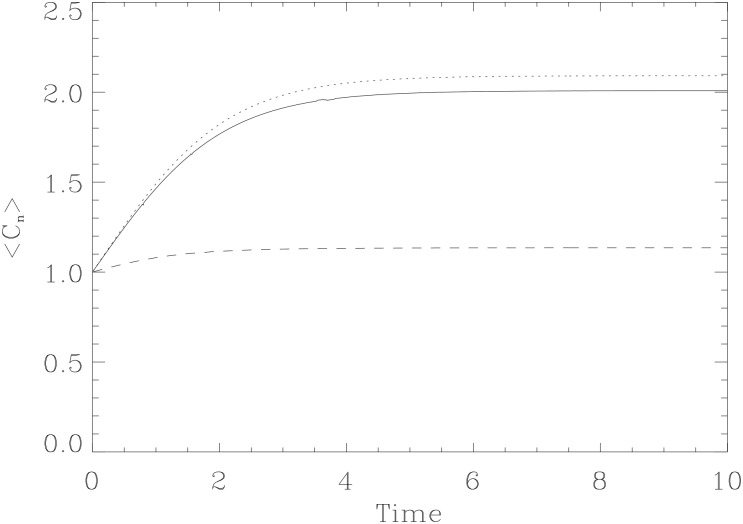
The time evolution of 〈*C*_*n*_〉. Time evolution of 〈*C*_*n*_〉 corresponding to the three cases in Fig 2(A), 2(B) and 2(C) shown in solid, dotted and dashed lines, respectively.

For our non-linear model Eq 8, it is interesting to find the average population 〈*C*_*n*_〉 in the limits of large and small *β*, corresponding to small and large stochastic noises *g*_*c*_ (small and large *D*), respectively. First, in the limit of *β* → ∞, we can show that

$$\langle C_{n}\rangle \to \gamma /\epsilon ,$$

which recovers the result for a deterministic logistic saturation where *C*_*n*_ saturates to its carrying capacity C_*_ = *γ*/*ϵ* (Fig 3) (additional derivations can be found in S1 Text). Mathematically, this is expected for logistic functions as the loss of higher-order non-linear terms leaves just the first-order exponential component. Intuitively, this relationship demonstrates the expected property of boundless growth due to a derepression of a coherent auto-inhibition of growth.

In the opposite limit of *β* → 0,

$$\left\langle C_{n}\right\rangle \propto -\sqrt{\frac{\beta }{\pi }}\ln[\frac{\epsilon^{2}}{\gamma D}].$$

**Fig 4:**
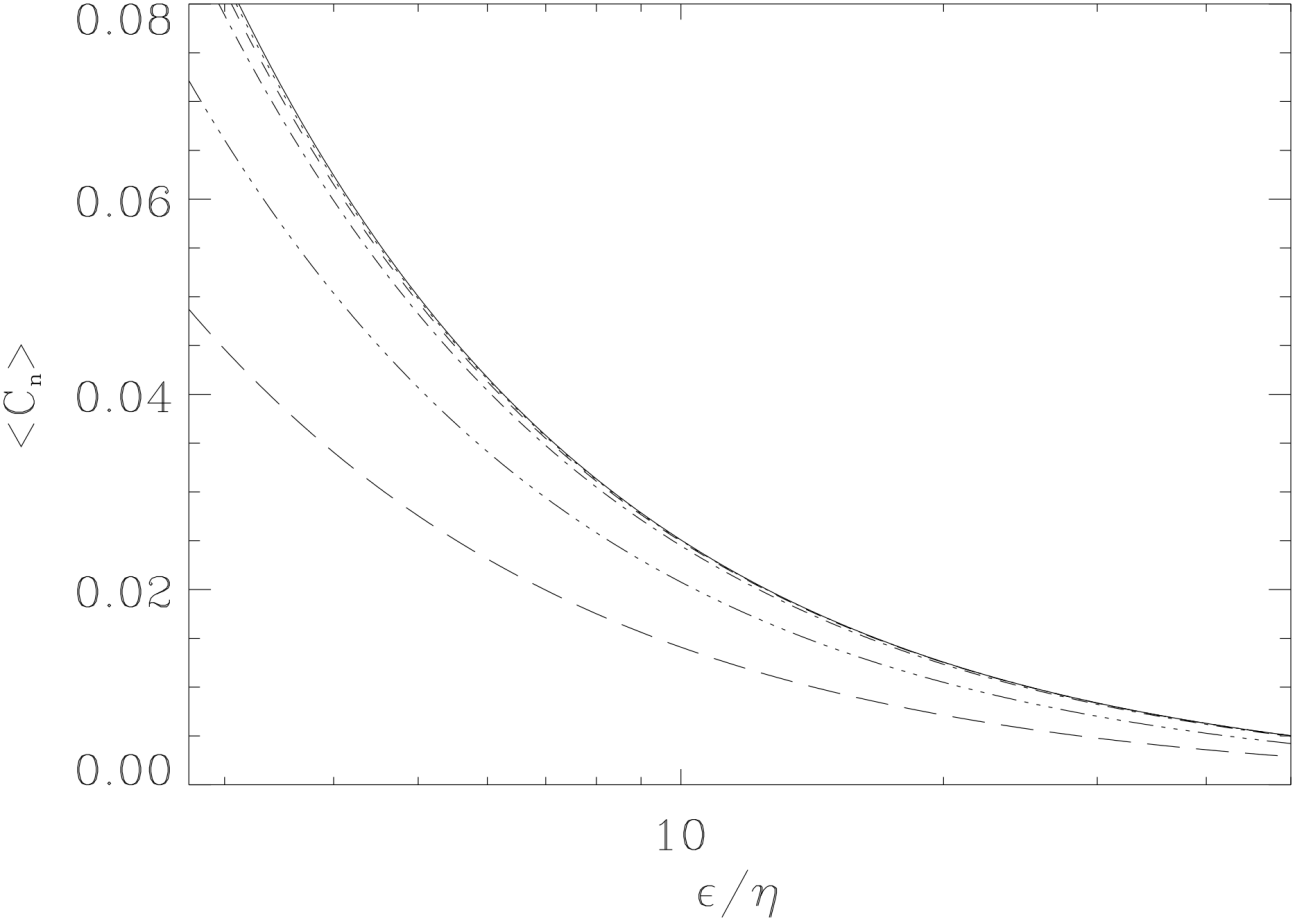
High- and low-*β* limits for ⟨*C*_*n*_⟩. Various trajectories for ⟨*C*_*n*_⟩ for increasing *ϵ*/*η* plotted for various *β* at *γ* = 0.5. Shown are *β* values where *β* = 10^*n*^ (*n* = −0.5, 0, 0.5, …, 2.0). Trajectories of ⟨*C*_*n*_⟩ approach a limit as *β* increases from small *β* = 10^−0.5^ (long dashes) to large *β* = 10^2^ (right-most solid).

Thus, as the coherent self-regulation vanishes as *ϵ* → 0, the average population 〈*C*_*n*_〉 diverges logarithmically. This behavior can be seen from Fig 4, where the value of 〈*C*_*n*_〉 becomes very large as *ϵ* becomes small, reflecting the fact that stochastic self-regulation alone cannot limit the population to a finite value. This also leads to unbounded growth as previously noted.

### 1.3 Implications

#### Negative Growth Rate

Our analysis above demonstrates not only that random noise with zero average can drive exponentially growing populations (linear model), but also that populations starting from the same initial conditions generate populations of differing size, with rapidly increasing intermittency in time. In addition to a purely random noise *g*_*w*_, when the growth rate also has a constant part *γ* (with the total growth rate *γ* + *g*_*w*_), the constant value *γ* can simply be added to Eqs (19) and (20). Specifically, Eq (19) becomes

$$\left\langle C_{l}\right\rangle \propto e^{(\gamma +\alpha )t},\;\;\;\Delta C_{l}\propto e^{(\gamma +2\alpha )t}.$$ (21)

The implication of Eq (21) is that 〈*C*_*l*_〉 can grow even when *γ* is negative as long as ∣*γ*∣ < *α* due to the growth of the large population. Furthermore, even when ∣*γ*∣ < *α* with a total negative growth rate *γ* + *α* < 0, the dispersion Δ*C*_*l*_ can still grow exponentially as long as ∣*γ*∣ < 2*α*. That is, colonies of large population size can grow even when the average growth rate is negative.

#### Adjustable Variance

Another interesting consequence is that the average growth rate remains the same as long as the total growth rate *γ* + *α* is the same regardless of the value of stochasticity (*α*). To illustrate this, let us consider a population *C*_*L*_ driven by a constant *γ*_*L*_ and stochastic growth rate with *α*_*L*_ where *α*_*L*_ = 0.01*α* and *γ*_*L*_ = *γ* + 0.99*α*. For *C*_*L*_, Eq (21) becomes

$$\left\langle C_{L}\right\rangle \propto e^{(\gamma_{L}+\alpha_{L})t}=e^{(\gamma +\alpha )t},\;\;\;\Delta C_{L}\propto e^{(\gamma_{L}+2\alpha_{L})t}=e^{(\gamma +1.01\alpha )t}.$$ (22)

Therefore, *C*_*l*_ and *C*_*L*_ grow at the same rate on average while *C*_*L*_ has a much smaller dispersion than *C*_*l*_, meaning that *C*_*L*_ has a much narrower distribution than *C*_*l*_. An example of such case of *C*_*L*_ is illustrated in Fig 1c where *D* = 1 and *τ*_*c*_ = 0.01, to be compared with the distribution of *C*_*l*_ in Fig 1a.

#### Growth Rate Variability

To understand the relationship between growth rate variability and population size, it is useful to examine how growth rate changes in relation to population. The growth rate of the average population 〈*C*_*n*_〉 at time *t* is given by

$$\frac{1}{\left\langle C_{n}\right\rangle }\frac{\partial \langle C_{n}\rangle }{\partial t}=\frac{1}{\int_{0}^{\infty }\;dC_{n}C_{n}P\left(C_{n},t\right)}\;\int_{0}^{\infty }\;dC_{n}C_{n}\frac{\partial P(C_{n},t)}{\partial t}.$$ (23)

To obtain the growth rate at a specific population, rather than for average populations, we consider the average of *C*_*n*_ over a small interval *dC*_*n*_ ≪ 1 around *C*_*n*_ and define a local growth rate *χ*(*C*_*n*_, *t*) at *C*_*n*_ and *t*:

$$\chi \left(C_{n},t\right)=\frac{1}{P(C_{n},t)}\frac{\partial P(C_{n},t)}{\partial t}.$$ (24)

From Eqs (24) and (18), we then obtain

$$\begin{array}{ccc} \chi (C_{n},t) & ∼ & \frac{\gamma e^{-2\gamma t}}{1-e^{-2\gamma t}}\left[-1+2\beta \;{\left[\frac{1}{C_{n}}-\frac{e^{-\gamma t}}{C(0)}-\frac{\epsilon }{\gamma }\left(1-e^{-\gamma t}\right)\right]}^{2}\right]\\ & & -2\beta \;[\frac{1}{C_{n}}-\frac{e^{-\gamma t}}{C(0)}-\frac{\epsilon }{\gamma }\left(1-e^{-\gamma t}\right)]\;[\frac{\gamma }{C_{0}}-\epsilon ]\;e^{-\gamma t}. \end{array}$$ (25)

**Fig 5:**
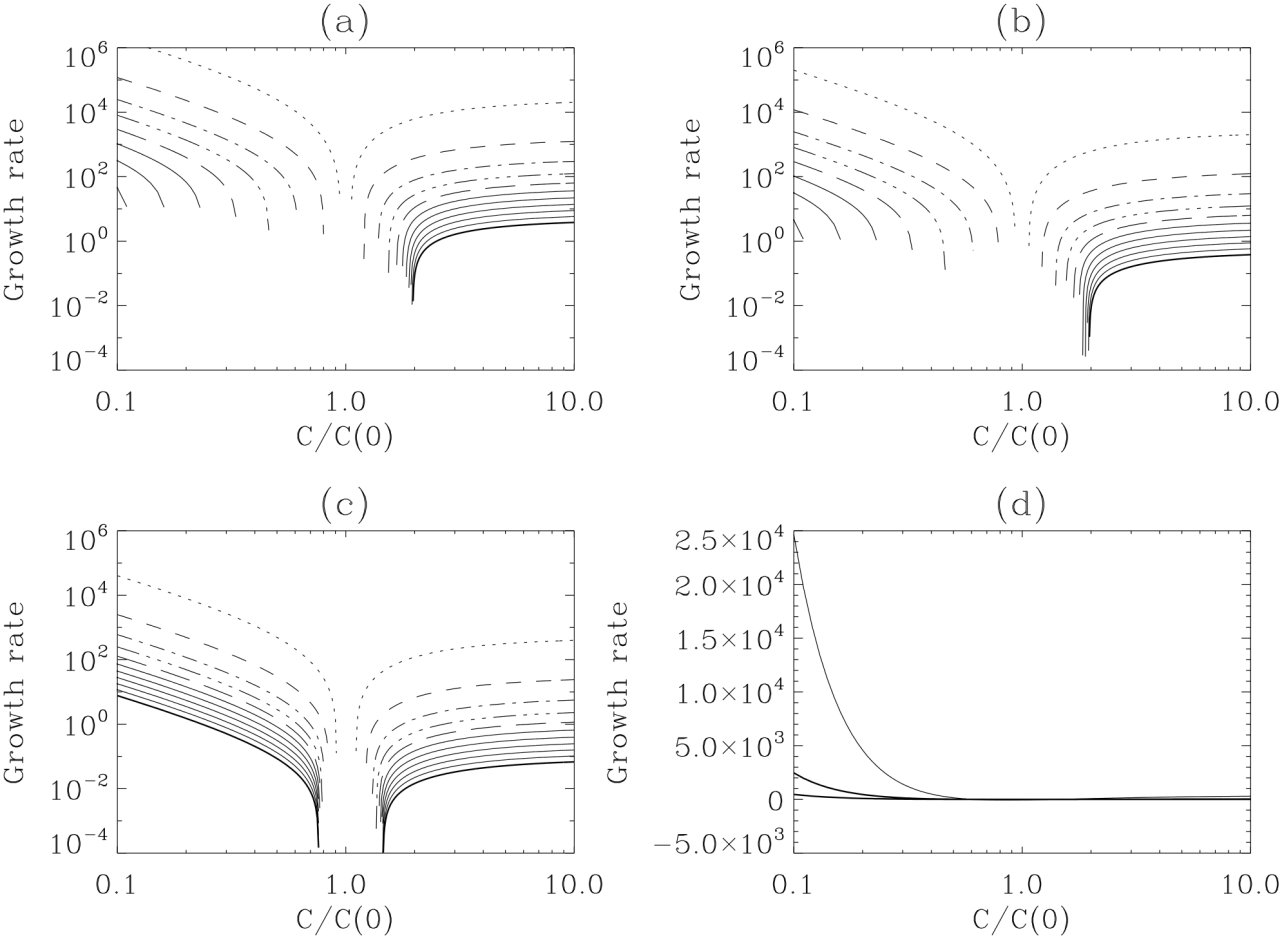
Growth rate variability of *C*_*n*_. Local growth rates of *χ*(*C*_*n*_, *t*) corresponding to the three cases in Fig 2(A), 2(B) and 2(C) shown in solid, dotted and dashed lines, respectively. Negative growth rates are not shown in panel (A)-(C). *χ*(*C*_*n*_, *t*) at *t* = 0.8 in panel (A), (B), and (C) are plotted together in panel (D).

We plot a local growth rate *χ*(*C*_*n*_, *t*) as defined in Eq (25) in Fig 5 for the cases shown in Fig 2. Specifically, Fig 5a–5c show snap-shots at different times, with time increasing from the top to the bottom lines; the negative growth rate at small *C*_*n*_ is not shown. *χ*(*C*_*n*_, *t*) at *t* = 0.8 in panel (a), (b) and (c) are plotted together in panel (d). We observe from Fig 5(a)–5(c) that *χ* decreases in time, even becoming negative for small *C*_*n*_, until *χ* becomes zero. Specifically, after a sufficiently long time (say, *t* > 4), *χ*(*C*_*n*_, *t*) becomes zero at all *C*_*n*_ as the PDFs converge to stationary profiles. Interestingly, in all cases, for *C*_*n*_ < C_*_, *χ* decreases with *C*_*n*_, in agreement with the data [15, 16]. For *C*_*n*_ > C_*_, the value of *χ* is very small with the tendency of a slight increase with *C*_*n*_. Our results thus clearly illustrate the strong variability of growth rate with respect to differing population size.

### 1.4 Fitness

We propose that under a known fitness function describing probability of survival, we can recast the problem of evolutionary selection as an optimization/maximization problem in the context of clonal phenotypic variability. In particular, by modeling clonal populations as existing as a collection of stochastically varying sub-populations, such as in a collection of isogenic yeast colonies, we can mathematically describe how individual sub-populations may yield differential responses to some defined selection sweep. Such differential responses have been previously described in the literature, such as with the phage-*λ* [2] or *B. subtilis* [3]. These responses have been generally described as belonging to a set of binary phenotypes which inherently confer differing evolutionary advantages. With the introduction of a fitness function, we introduce two binary phenotypes—that of death and survival.

**Fig 6:**
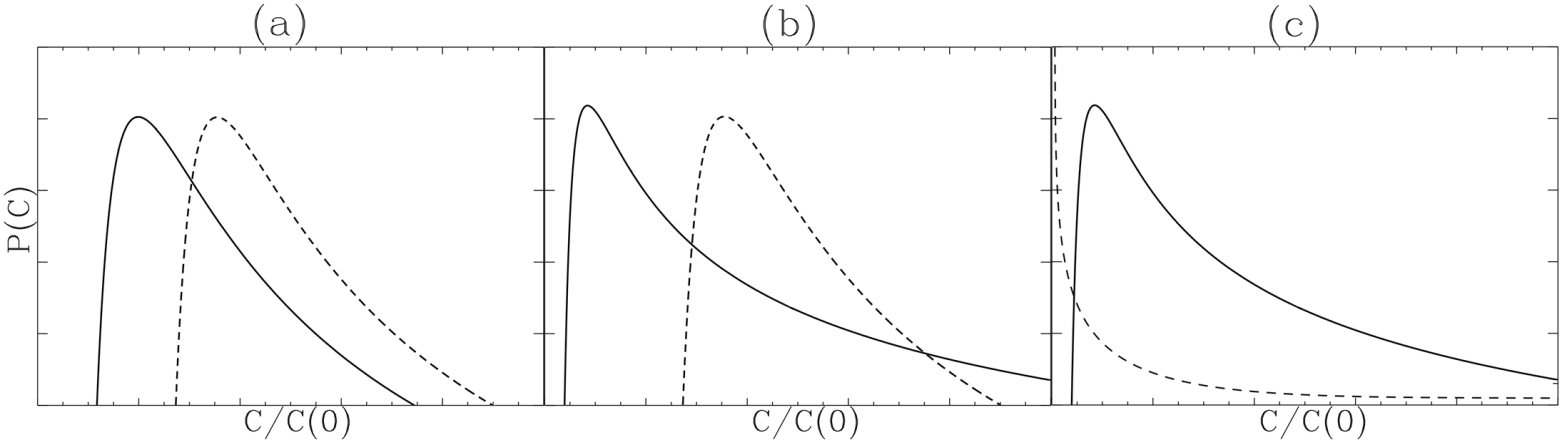
Evolution under noisy selection regimes. Initial distributions (solid line) and fitness functions (dotted line) are plotted for various selection regimes. Panel (A) is representative of a directed (positive) selection regime, panel (B) is representative of a stabilizing selection regime, while panel (C) is representative of a selection regime representative of therapeutic treatment.

Consider a collection of co-cultured isogenic yeast colonies beginning in identical initial conditions. Due to stochastic fluctuations in growth rate, each colony begins to adopt unique growth trajectories thereby increasing the accessible state-space of the collection of colonies. At some later time, a toxic substance is introduced into the media. Intake of this toxic substance would be a function of the total surface area in contact with the media (surface area $\propto C^{{}^{2}\;{/}_{3}}$) thereby relating increasing colony size to increasing risk of death. Therefore, the probability of survival may be quantified as some function of colony size, *f*(*C*) (Fig 6). As *f*(*C*) is a probability distribution function, the probability of survival after selection for populations in a state on [*C*, *C* + *dC*] is given by *f*(*C*)*P*(*C*)*dC*. In the context of such a fitness function, we hypothesize that under repeated application of identical selection pressure the net genetic changes, reflected by our model parameters *τ*_*c*_, *ϵ*, *γ*, etc., will be to maximize the total fraction of survivors after selection, described by the survivability coefficient *S*, where

$$S=\int_{0}^{\infty }\;f\left(C\right)P\left(C\right)dC.$$ (26)

## 2 Conclusion and Discussion

### 2.1 Evolution

In [17], it was proposed that an increase of epigenetic variability in phenotype, as opposed to a shift in the mean phenotype, could be a driving force of evolutionary adaptation. A similar view has been proposed that such mechanisms could be a crucial mechanism for evolutionary processes in cancer [5]. Here we present a mathematical formalism, in the form of the survivability coefficient, that reveals how such an increase of phenotypic variability may affect evolutionary processes by linking cellular survival and biological noise. While it is yet to be determined whether such evolutionary tuning via changes in variability may be the result of heritable genetic variation as presented in [17] or the result of non-heritable processes as presented in [5], we demonstrate here that stochastic processes alone can provide the mechanism for adjusting phenotypic variability. Moreover, a unification between both paradigms can be achieved by simply considering a mixture of stochastically evolving systems occupying multiple points in parameter-space.

While our probabilistic approach to survival in the context of phenotypic heterogeneity provides a key alternative paradigm for understanding evolutionary processes, rigorously defined models of stochastic processes via intrinsic chemical noise [6, 18] have led to many other successful predictions of biological phenomena. Such predictions based upon molecular fluctuations generally attempt to predict binary phenotypes that emerge probabilistically on a macroscopic scale [2, 3]. In contrast, we present a more nuanced perspective of phenotypic heterogeneity that, with the application of a probabilistic fitness function, can yield a differential evolutionary response through our examination of the effects of stochasticity. Despite the Poissonian nature of microscopic processes, stochastic behavior of a variety of types can emerge at a macroscopic level, such as the geometric distribution of translational noise in prokaryotes [19]. However, as our model is not directly based on molecular counts or chemical concentrations, the source of stochasticity is thus not limited to only chemical noise—our aim was to explore the implications of stochasticity via the action of a randomly (Gaussian) distributed growth rate utilizing a probabilistic methodology. As described in [1], protein levels of individual cells can vary drastically with auto-correlation characteristics similar to Eq (2), which can be ascribed to inherent variability associated with upstream regulatory components as well as transcriptional and translational bursting [1, 20]. Instead, we present a phenomenological model of noise-driven growth that reveals deeper scientific implications of the relationship between stochasticity and cellular heterogeneity.

A key factor in the model we have presented is the finite correlation time (*τ*_*c*_), which is related to the idea of cellular memory [1, 9, 21]. Each sub-population (individual iterations of *C*_*l*_ or *C*_*n*_) acts independently of another, while the driving noise measured of at any two time points yield identical, but not independent, distributions depending on the degree of auto-correlation. With increased auto-correlation, intermittency decreases, as fewer fluctuations occur in the time-course of individual sub-populations. In that case, fast growing sub-populations will tend to remain in a fast-growing state, while slow growers will similarly tend to remain in a slow-growing state. This, in essence, increases the overall variance of the entire population (the ensemble of all sub-populations), thereby increasing the chances of producing survivors under repeated, identical selection events. Similarly, having a shorter auto-correlation time allows sub-populations to more rapidly switch states (higher intermittency). However, this generates an evolutionary trade-off, as a re-population of formerly occupied states are regained quickly, but a smaller overall area of state-space will be sampled.

To the extent that a stochastic growth rate and the loss of coherent self-regulation can drive phenotypic variability, such de-regulation can be seen as a process of decanalization. According to C. H. Waddington, canalization describes the phenomenon where, “the genotype can, as it were, absorb a certain amount of its own variation without exhibiting any alteration in development.” [22]. Roughly speaking, we can consider canalization as the process by which complex gene regulatory networks, when selected for developmental stability, become insensitive to genetic mutations with respect to phenotype [23]. We present here an alternative mathematical framework of canalization where a particular genotype is indicative of a particular set of parameters, such as *τ*_*c*_ or *ϵ*. Given a series of mutations that decreases coherent self-regulation (decreased *ϵ*), and increases self-regulatory stochasticity (increased $\left\langle g_{c}^{2}\right\rangle$), the subsequent phenotypic variability will, according to our model, increase (increased Δ*C*_*n*_).

The non-linear model brings out the essential effects of a loss of coherent self-regulation by multiplicative noise. That is to say, the lack of such a self-regulation mechanism yields boundless growth which may be reversed with the implementation of a self-regulation mechanism. Such self-regulation, which may be variously coherent or stochastic, induces convergence to a stationary steady-state distribution while still allowing for heterogeneity on an individual level. Unlike in the un-regulated case, there are constraints on which states will likely be accessed as defined by the steady-state distribution. This property remains true even in the case of the total loss of coherent self-regulation, where coherent self-regulation is replaced with a wholly stochastic self-regulation. The net effect of stochastic self-regulation is a subsequent expansion of the allowable state-space of the isogenic population.

The models can also provide a mechanism for the transient entrance of bacterial subpopulations into a persister state. When our models are compared to distributions of persister and replicator states (c.f. [24]), we find that we can explain certain features of these distributions. In particular, we observe that the distribution of persisters (no growth) is less skewed than the distribution of replicators (high growth). This is indicative of the slow growing distribution having smaller mean growth as well as having a smaller fraction of large subpopulations (high *C*_*n*_ values). Similarly, the distribution for fast growers have a larger fraction of large subpopulations. This set of observations are consistent with our model in that distributions from parameter sets with smaller mean growth are more Gaussian and less intermittent in nature.

### 2.2 Cancer

One motivation for the development of this model was to propose a mathematical framework within which we can understand resistance to treatment under the Frank-Rosner paradigm of drug resistance [5]. While most previous work on tumor growth modeling [25–33] has largely focused on the deterministic growth of tumors for mean values, heterogeneity and variability are key factors in understanding the development of tumors [5, 34, 35]. It has been observed that cancerous cells have greater variability in growth rate than do normal cells [36], confirming that there is merit to the assumption that growth rate is driven by a random process. In particular, we propose that stochasticity in growth rate and a loss of coherent self-regulation can provide the basis for the mechanism of short-range search as described in [5]. Randomly fluctuating elements within identical cells can produce a range of responses to any particular stimulus. As described by Frank and Rosner, differential responses would, therefore, be expected to any particular therapeutic drug due to phenotypic variability. For example, given a correlation between a sub-population’s surface area and that sub-population’s metabolic uptake, and given that a particular cancer therapeutic can induce lethality only in cells with metabolic uptake above a certain threshold, there would always remain a non-zero probability that one or more sub-populations below that threshold exists and are, according to our model, resistant to treatment (i.e. tumor size too small to sufficiently induce lethality). Beginning with that sub-population’s resistant state, a new population can be derived, using that resistant sub-population’s current state as a new initial condition. In this context, phenotypic variability within isogenic populations provide a buffering, or bet-hedging, against dramatic fitness events, such as the introduction of a cancer drug into the body or sudden heat-shock. In contrast with the standard deterministic approaches utilized in mathematical models of cancer growth, our stochastic cancer growth model provides both a mechanism for cellular bet-hedging as well as the dynamics following a dramatic selection sweep. Bet-hedging phenomena, similar to that described above, have been demonstrated and studied in yeast [4], while recent efforts [37] have begun to explore the relationship between heterogeneity and drug-resistance.

An important feature of the model regards the limits of the mean value for our models. The linear model shows that the mean population value is exponentially increasing. However, interestingly the non-linear model shows two regimes of stability. In particular, in the large *ϵ* regime, the mean population value results in a logistic growth where the mean value reaches a steady-state distribution. In contrast, in the small *ϵ* regime, the mean population value grows in an unbounded manner. We predict that the transition between a normal and cancerous state in any given tissue may be accompanied by a set of genetic or epigenetic changes that will lead to a transition of the population from a large *ϵ* to a small *ϵ* regime.

The results of our model have specific implications for designing a potential treatment regimen for cancer patients. The first consideration involves the overall variance of a heterogeneous cancer population while the second is in regards to the role of intermittency in particular cancer sub-populations. We must first caution that while the predictions of our model may have potential therapeutic implications, the level of detail presented in our models remains at a wholly phenomenological level. As such, given a future progression towards more chemically oriented models, the predicted therapeutic implications could be provided at a higher level of detail.

It may be possible to increase the efficacy of a given treatment by engaging a patient with one or more neo-adjuvant therapies with the goal of reducing the overall phenotypic variability of all tumor sub-populations. It is important to note that the purpose of this neo-adjuvant therapy would *not* be to reduce the volume of a tumor (summation over all sub-populations), but instead to reduce the heterogeneity and phenotypic variability of individual subpopulations (Δ*C*_*n*_). This reduction of phenotypic variance would thereby reduce the probability that any sub-populations would survive a given cancer treatment, essentially by reducing the action of cellular bet-hedging. Just as serum-starvation can increase the efficiency of an experimental protocol by synchronizing cells to the same stage of the cell cycle, this neo-adjuvant therapy need not be a cancer therapeutic—it would simply need to reduce the overall non-genetic heterogeneity of all cancer cells.

The second implication of our model for treatment involves the role of intermittency in treatment. Assuming we begin the post-treatment period with a collection of residual sub-populations that survived treatment, the state-space available to these sub-populations is limited by the degree of intermittency that is inherent to those cells’ genotype. In particular, this intermittency is defined by the auto-correlation property (*τ*_*c*_) of the noise described in our model. The auto-correlation property serves to reflect, roughly, how rapidly a given system may change its random rate through time. In this post-treatment scenario, it serves to effectively limit the accessible state-space for surviving tumor sub-populations. For example, if the surviving sub-populations demonstrated low auto-correlation immediately following treatment, these surviving sub-populations could rapidly transition into a state where they are susceptible to a second round of treatment. Alternatively, if the surviving sub-populations demonstrated high auto-correlation, these other surviving sub-populations are likely to stay in a treatment-resistant state, as they were already in a treatment-resistant state immediately following treatment. As the time to transition into a treatment-susceptible state is, on average, longer for sub-populations with higher auto-correlation, further treatment for highly auto-correlated tumors would likely be ineffective until the tumor regains its former levels of heterogeneity.

## Supporting Information

S1 Text Computational Validation. Details on the computational validation of solutions are presented.
(PDF)

S1 Fig Auto-correlation of noise as implemented by the numerical solver. Shown are calculated correlation values for 〈*g*_*c*_(*t*)*g*_*c*_(*t*′)〉 = *D* exp [−∣*t* − *t*′∣/*τ*_*c*_] with *τ*_*c*_ = *e* ^*n*^ where *n* = 1 (leftmost curve), 2, 3, 4, and 5 (rightmost curve). Ensembles consisted of 1000 replications with time steps of 0.1 seconds.
(TIFF)

S2 Fig Individual solution trajectories. Three individual solutions are shown above for times between 0 and 1, where *τ*_*c*_ = 0.01 and *D* = 100. As evident in the figure, individual solutions adopt differing and independent trajectories.
(TIFF)

S3 Fig Computational validation of probability distribution functions of *C*_*l*_. Three PDFs are shown above for times *t* = 0.05, 0.15, and 0.25 seconds, where *τ*_*c*_ = 0.01 and *D* = 100. The model presented here is described by Eq 3 of the main text. The empirical histograms are plotted and matching analytical solutions are overlaid in red, dashed lines.
(TIFF)

S4 Fig Computational validation of probability distribution functions of *C*_*n*_. Three PDFs are shown above for times *t* = 0.05, 0.2, and 0.35 seconds, where *τ*_*c*_ = 0.01, *D* = 100, *γ* = 1, and *ϵ* = 0.5. The model presented here is described by Eq 8 of the main text. The empirical histograms are plotted and matching analytical solutions are overlaid in red, dashed lines.
(TIFF)
