# Supplementary material for: Noise-Driven Phenotypic Heterogeneity with Finite Correlation Time in Clonal Populations
Source: PLoS One. 2015 Jul 23;10(7):e0132397. doi: 10.1371/journal.pone.0132397 (PMC4512695; doi:10.1371/journal.pone.0132397)
Supplement: S1 Text — Details on the computational validation of solutions are presented. (PDF) [file pone.0132397.s001.pdf]

I. STOCHASTIC RUNGE-KUTTA ALGORITHM

To validate the analytical preditions presented in the main manuscript, a numerical approach was utilized. As the source of noise was modeled as having a non-delta auto-correlation function, special consideration must be taken to ensure that all numerical simulations replicate this property. To that end, a second order stochastic Runge-Kutta algorithm that demonstrates a similar exponential auto-correlation property was utilized. [1]. This algorithm solves a paired set of stochastic differential equations that produces the desired auto-correlation property. (See Supplemental Figure 1)

As the models presented are in the form of stochastic differential equations, individual solution trajectories may vary greatly (See Supplemental Figure 2). Therefore, to validate the analytically derived probability distribution functions (PDFs), a statistical ensemble of solutions were generated for each parameter set. Unless otherwise noted, all ensembles consisted of 50,000 individual solutions, $C_0 = 1$, and time steps of 0.001 seconds. Given these statistical ensembles, histograms of numerically obtained distributions were compared to their corresponding analytical solutions (See Supplemental Figures 3, 4). All simulations were performed using compiled C++ code on Intel architecture. Histograms and analytical solutions were produced using R.

II. ADDITIONAL DERIVATIONS

A. C_l

We compute the average of C_l by using $\Gamma = \ln(C_l/C_0)$ (i.e. $C_l = C_0 e^{\Gamma}$) and thus $dC_l = d\Gamma C_l = d\Gamma C_0 e^{\Gamma}$ as follows:

$$\begin{aligned}
\langle C_l \rangle &= \int dC_l C_l p(C_l) \\
&= \int dC_l \frac{1}{2\sqrt{\alpha t \pi}} \exp\,[-\frac{[\ln C_l/C_0]^2}{4\alpha t}] \\
&= \frac{1}{2\sqrt{\alpha t \pi}} \int d\Gamma C_0 \exp\,[\Gamma] \exp\,[-\frac{\Gamma^2}{4\alpha t}] \\
&= \frac{1}{2\sqrt{\alpha t \pi}} C_0 \int d\Gamma \exp\,[-\frac{[\Gamma - 2\alpha t]^2}{4\alpha t}] \exp\,[\alpha t] \\
&= C_0 e^{\alpha t} \qquad (1)
\end{aligned}$$

For the dispersion ΔC_l, we compute $\langle C_l^2 \rangle$ following the similar steps as above:

$$\begin{aligned}
\langle C_l^2 \rangle &= \int dC_l C_l^2 p(C_l) \\
&= \int dC_l C_l \frac{1}{2\sqrt{\alpha t \pi}} \exp\,[-\frac{[\ln C_l/C_0]^2}{4\alpha t}] \\
&= \frac{1}{2\sqrt{\alpha t \pi}} \int d\Gamma C_0^2 \exp\,[2\Gamma] \exp\,[-\frac{\Gamma^2}{4\alpha t}] \\
&= \frac{1}{2\sqrt{\alpha t \pi}} C_0^2 \int d\Gamma \exp\,[-\frac{[\Gamma - 4\alpha t]^2}{4\alpha t}] \exp\,[4\alpha t] \\
&= C_0^2 e^{4\alpha t}
\end{aligned} \tag{2}$$

Thus,

$$\Delta C_l = \sqrt{\langle C_l^2 \rangle - \langle C_l \rangle^2} = C_0 \sqrt{e^{4\alpha t} - e^{2\alpha t}} \approx C_0 e^{2\alpha t}, \tag{3}$$

where again $C_0 = C(t = 0)$ is the initial value of C at $t = 0$.

B. C_n

It is interesting first to find out the average population $\langle C_n \rangle$ in the limits of large and small β, corresponding to small and large stochastic noises g_c, respectively. To obtain the mean value of C_n, we use

$$\begin{aligned}
\langle C_n \rangle &= \int_0^\infty dC_n P[C_n] C_n \\
&= \int_{-A}^\infty dz P[z] C_n \\
&= \int_{-A}^\infty dz P[z] \frac{1}{z + A} \\
&= N \int_{-A}^\infty dz \exp\left[-\beta z^2\right] \frac{1}{z + A}.
\end{aligned} \tag{4}$$

where $N^{-1} = \int_{-A}^\infty dz P[z]$ and $A = e^{-\gamma t}/C(0) + \frac{\epsilon}{\gamma}[1 - e^{-\gamma t}]$: Note that we used $dC_n P[C_n] = dz P[z]$ and the formulation for $\langle z^2 \rangle$.

First, in the limit of $\beta \to \infty$ ($\xi \to 0$), we evaluate Eq. (4) to leading order in $1/\beta$ by

using the variable $w = \sqrt{\beta} z$ as follows:

$$\begin{aligned}
\langle C_n \rangle &= N \int_{-\sqrt{\beta}A}^{\infty} \frac{dw}{\sqrt{\beta}} \exp\left[-w^2\right] \frac{1}{\frac{w}{\sqrt{\beta}} + A} \\
&\sim \sqrt{\frac{\beta}{\pi}} \int_{-\infty}^{\infty} \frac{dw}{\sqrt{\beta}} \exp\left[-w^2\right] \frac{1}{A} \\
&= \frac{1}{A} = \frac{1}{e^{-\gamma t}/C(0) + \frac{\epsilon}{\gamma}[1 - e^{-\gamma t}]},
\end{aligned} \tag{5}$$

where $N^{-1} \sim \sqrt{\frac{\pi}{\beta}}$ for $\beta \gg 1$ is used. In the limit of large $t \to \infty$, Eq. (5) simplies as

$$\langle C_n \rangle \to \gamma/\epsilon,$$

which recovers the result in a deterministic logistic system without a stochastic regulation ($\beta \to \infty$) where C saturates to its carrying capacity $C_* = \gamma/\epsilon$. This carrying capacity C_* obviously diverges when $\epsilon \to 0$ as a deterministic logistic system is solely governed by a linear term with an exponentially growing solution. This can be seen from Eq. (5) by taking $\epsilon = 0$: $\langle C_n \rangle = C(0) e^{\gamma t}$.

In the opposite limit of $\beta \to 0$ ($\xi \to \infty$) and $A \ll 1$, the integrand in Eq. (4) diverges at the low limit of the integration. To isolate the divergence, we let

$$\begin{aligned}
\langle C_n \rangle &= NI, \\
I &= \int_0^{\infty} dy \exp\left[-\beta(y - A)^2\right] \frac{1}{y} \\
&= \int_0^{\infty} dx \exp\left[-\phi(x - 1)^2\right] \frac{1}{x} \\
N^{-1} &= \int_{-A}^{\infty} dz \exp\left[-\beta z^2\right] \sim \frac{1}{2}\sqrt{\frac{\pi}{\beta}}
\end{aligned} \tag{6}$$

where $y = z + A$, $\phi = \beta A^2$, $x = y/A$, and $y(0) = 1/C(0)$ were used. Note that ϕ becomes small as β and A decrease, approaching

$$\phi \to \frac{\epsilon^2}{\gamma D}$$

for large time. As can be seen from above, when the coherent self-regulation (ϵ) is dominated by a linear growth (γ) and by the stochastic self-regulation (D) (i.e. as $\phi \to 0$), the integral for I in Eq. (6) can blow up. To isolate the divergence in I for small ϕ to leading order

in ϕ, we differentiate I with respect to ϕ to obtain the following differential equation for $I = I(\phi)$:

$$\begin{aligned}
\partial_\phi I &= -\int_0^\infty dx \frac{(x-1)^2}{x} \exp\left[-\phi(x-1)^2\right] \\
&= \int_0^\infty dx \left[(-x+2) - \frac{1}{x}\right] \exp\left[-\phi(x-1)^2\right] \\
&\equiv -G(\phi) - I. \qquad (7)
\end{aligned}$$

Here,

$$G(\phi) = \int_0^\infty dx (x-2) \exp\left[-\phi(x-1)^2\right]. \qquad (8)$$

Since $G(\phi)$ in Eq. (8) contains no singularity in the integrand as $\phi \to 0$, it can easily be computed to leading order in $\phi \ll 1$ by using $x = \chi\phi^{-1/2}$ as follows:

$$\begin{aligned}
G(\phi) &= \int_0^\infty dx (x-2) \exp\left[-\phi(x-1)^2\right] \\
&= \frac{1}{\phi}\int_0^\infty d\chi(\chi - 2\sqrt{\phi}) \exp\left[-(\chi^2 - 2\sqrt{\phi}\chi + \phi)\right] \\
&\sim \frac{1}{\phi}\int_0^\infty d\chi \chi \exp\left[-\chi^2\right] + O(\sqrt{\phi}) \\
&= \frac{1}{2\phi} + O(\sqrt{\phi}). \qquad (9)
\end{aligned}$$

Thus, by using $G(\phi)$ in Eq. (9) in the differential equation for $I(\phi)$ in Eq. (7), we obtain

$$\partial_\phi I + I \sim -\frac{1}{2\phi}, \qquad (10)$$

with the solution

$$I(\phi)e^{\phi} - I(\phi_0)e^{\phi_0} \sim -\frac{1}{2}\ln\left[\phi/\phi_0\right], \qquad (11)$$

where $\phi_0 \ll 1$ is the content value. Thus,

$$I(\phi) \sim -\frac{1}{2}[\ln\phi]e^{\phi} \sim -\frac{1}{2}\ln\phi \qquad (12)$$

to leading order in $\phi \ll 1$. Therefore, when $\phi \to 0$, $\langle C_n \rangle$ becomes

$$\begin{aligned}
\langle C_n \rangle &\sim -\sqrt{\frac{\beta}{\pi}}\ln\phi \\
&\sim -\sqrt{\frac{\beta}{\pi}}\ln\left[\beta A^2\right]. \qquad (13)
\end{aligned}$$

In the limit of large time $t \to \infty$, Eq. (13) becomes

$$\langle C_n \rangle \propto -\sqrt{\frac{\beta}{\pi}} \ln \left[\frac{\epsilon^2}{\gamma D} \right].$$

Thus, the RHS of Eq. (13) approaches $+\infty$ as $\epsilon \to 0$, leading to an unbounded growth.

To show how this unbounded growth proceeds in time, we evaluate Eq. (13) by using $A = 1/C_0 e^{\gamma t}$ for $\epsilon/\gamma = 0$ as:

$$\langle C_n \rangle \sim -\sqrt{\frac{\beta}{\pi}} \ln [\beta C_0^{-2} e^{-2\gamma t}] \sim \sqrt{\frac{\beta}{\pi}} 2\gamma t, \tag{14}$$

for $t \gg \ln (C_0/\sqrt{\beta})$ [as required for $\phi \ll 1$]. Thus, in the absence of the coherent self-regulation $\epsilon = 0$, the stochastic regulation leads to a linearly growing average population $\langle C_n \rangle$ in time. This unbounded growth reflects the fact that the stochastic self-regulation alone is not sufficiently effective in regulating growth to finite amplitude.

[1] R. Honeycutt, Phys. Rev. A, **45**, 604-610 (1992).
