# Supplementary figures and images for: Noise-Driven Phenotypic Heterogeneity with Finite Correlation Time in Clonal Populations
Source: PLoS One. 2015 Jul 23;10(7):e0132397. doi: 10.1371/journal.pone.0132397 (PMC4512695; doi:10.1371/journal.pone.0132397)

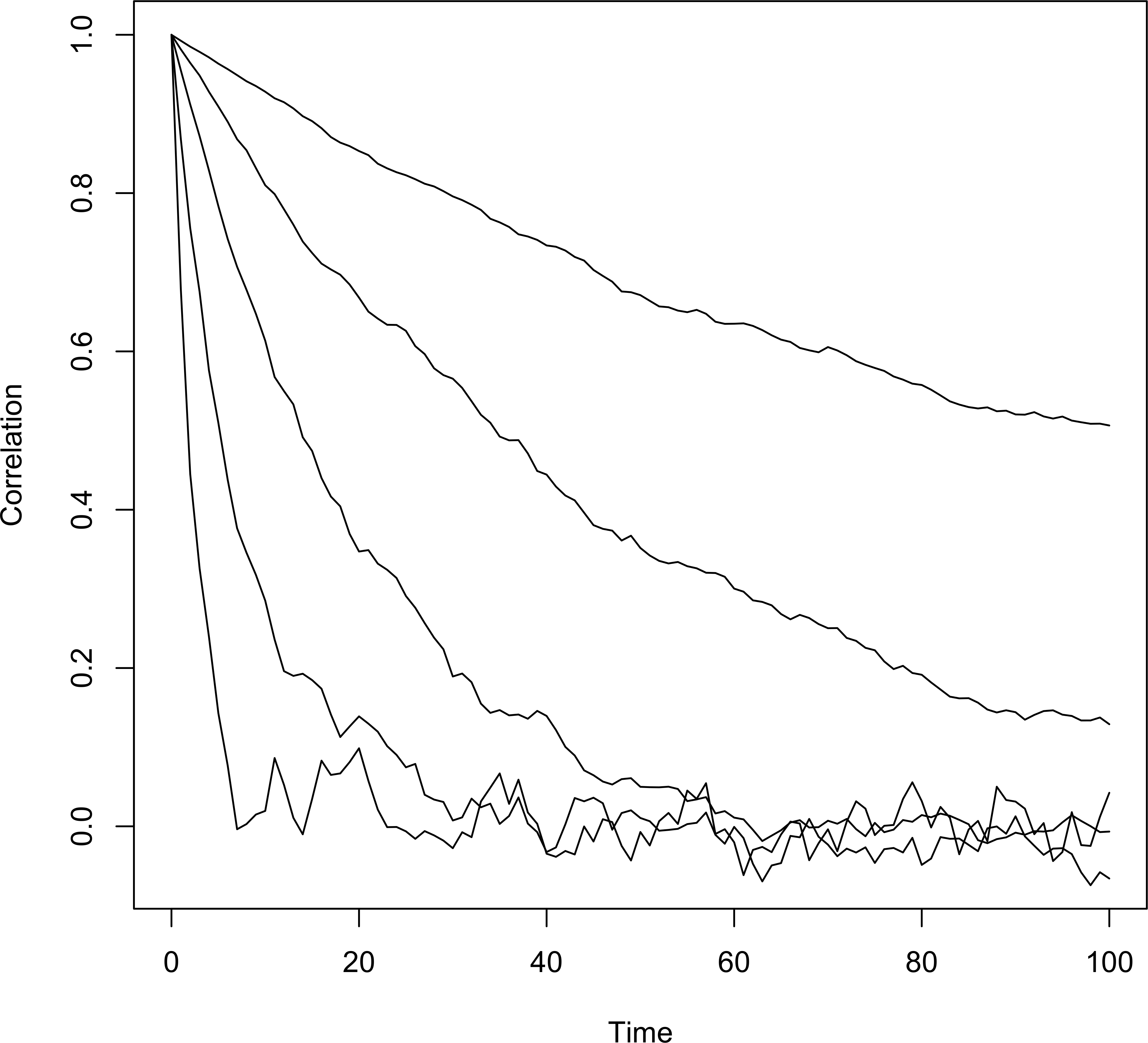

Supplement: S1 Fig — Shown are calculated correlation values for 〈g c(t)g c(t′)〉 = D exp [−∣t − t′∣/τ c] with τ c = e n where n = 1 (leftmost curve), 2, 3, 4, and 5 (rightmost curve). Ensembles consisted of 1000 replications with time steps of 0.1 seconds. (TIFF) [file pone.0132397.s002.tiff]

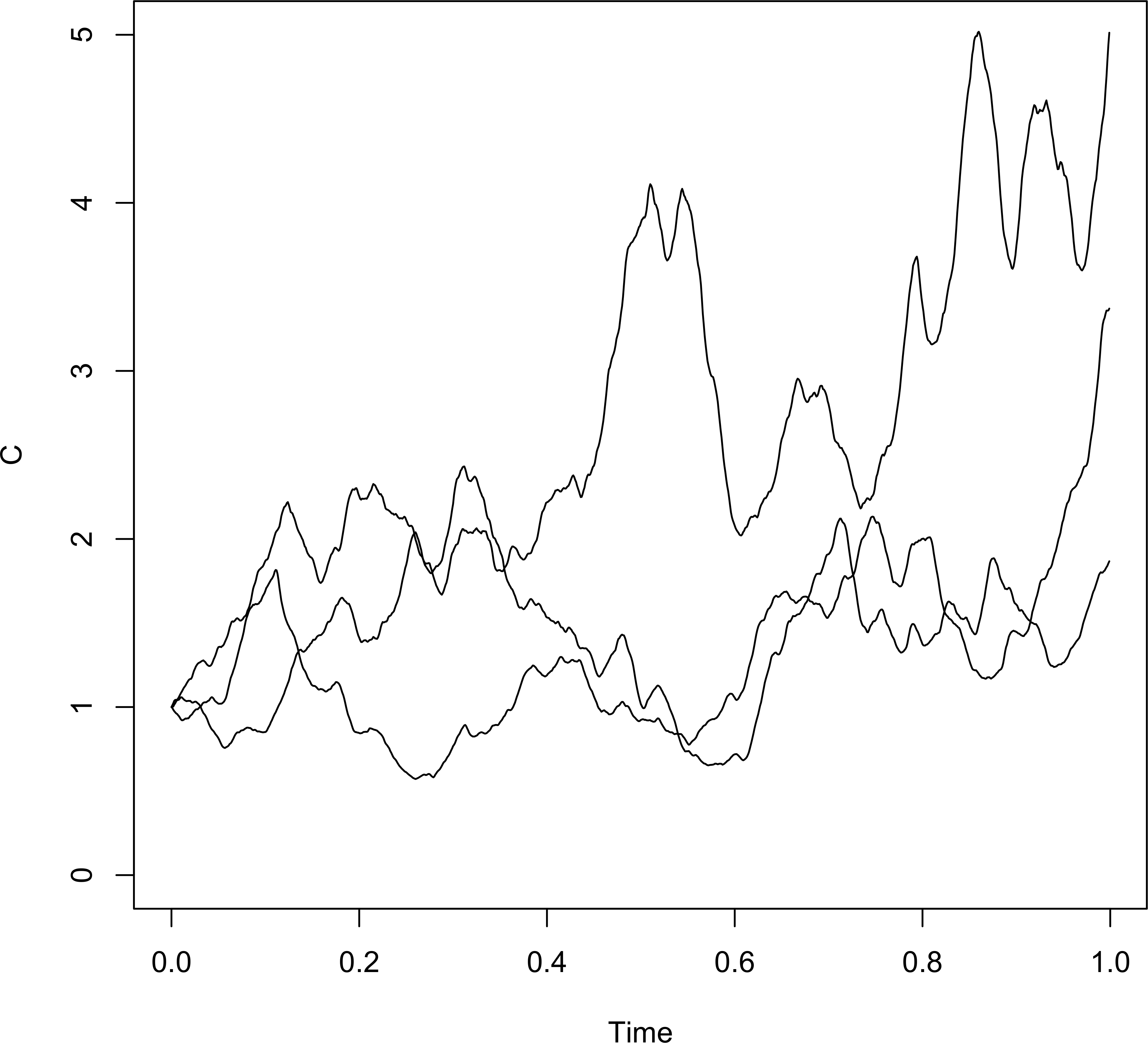

Supplement: S2 Fig — Three individual solutions are shown above for times between 0 and 1, where τ c = 0.01 and D = 100. As evident in the figure, individual solutions adopt differing and independent trajectories. (TIFF) [file pone.0132397.s003.tiff]

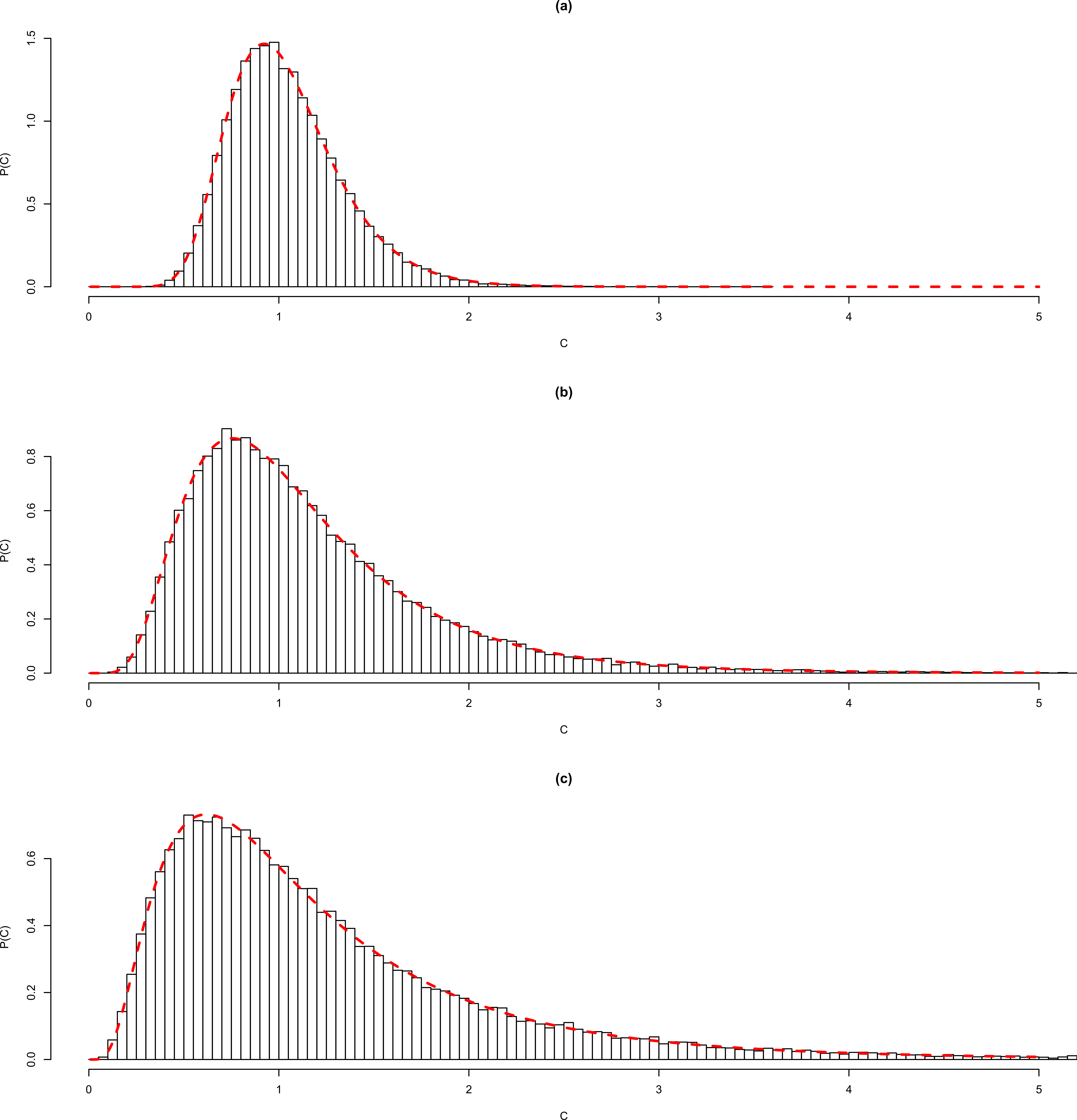

Supplement: S3 Fig — Three PDFs are shown above for times t = 0.05, 0.15, and 0.25 seconds, where τ c = 0.01 and D = 100. The model presented here is described by Eq 3 of the main text. The empirical histograms are plotted and matching analytical solutions are overlaid in red, dashed lines. (TIFF) [file pone.0132397.s004.tiff]

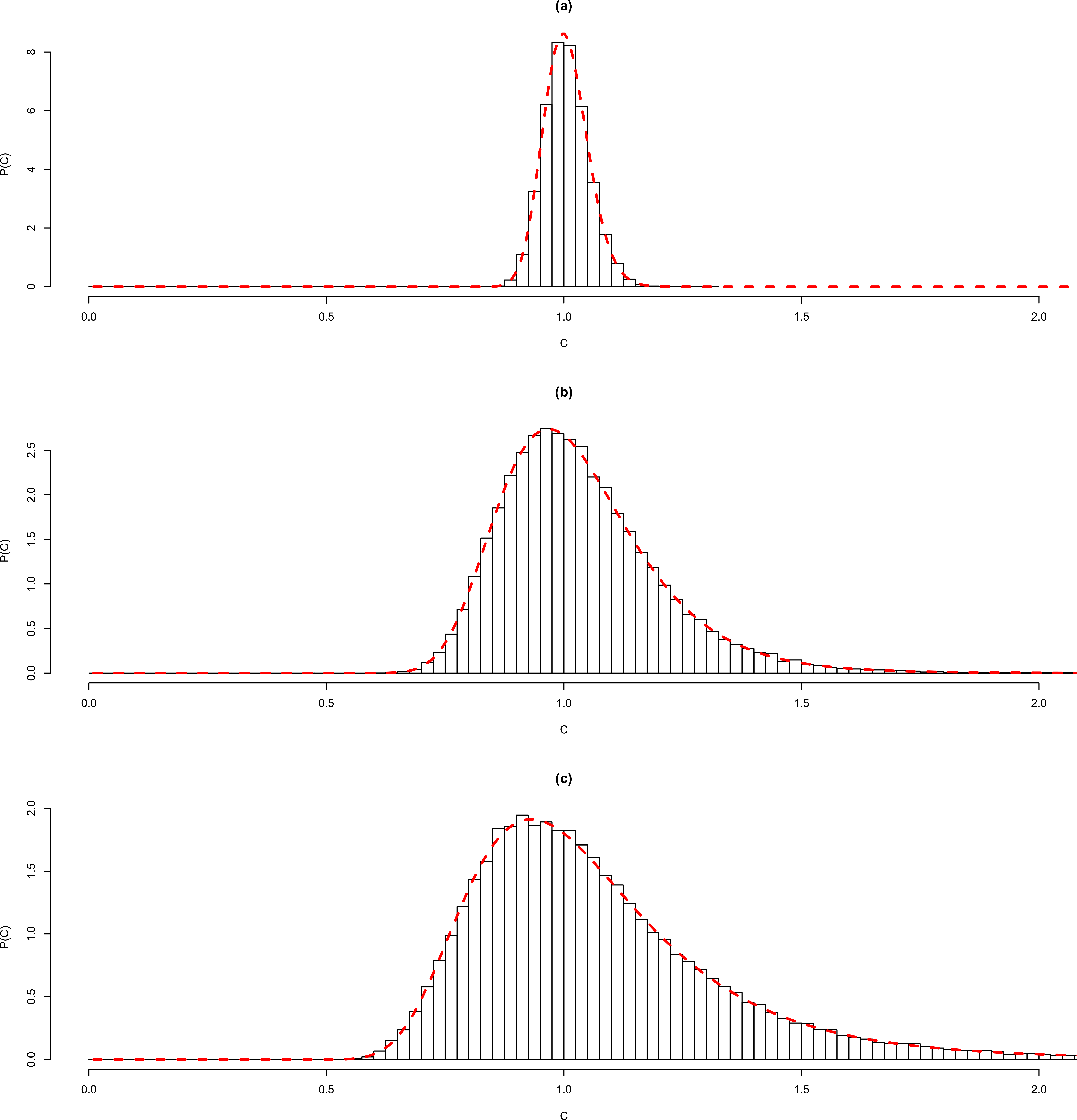

Supplement: S4 Fig — Three PDFs are shown above for times t = 0.05, 0.2, and 0.35 seconds, where τ c = 0.01, D = 100, γ = 1, and ϵ = 0.5. The model presented here is described by Eq 8 of the main text. The empirical histograms are plotted and matching analytical solutions are overlaid in red, dashed lines. (TIFF) [file pone.0132397.s005.tiff]
